# Predicting Body Weight in Pre-Weaned Holstein–Friesian Calves Using Morphometric Measurements

**DOI:** 10.3390/ani14142129

**Published:** 2024-07-21

**Authors:** Flávio G. Silva, Emanuel Carreira, Joana M. Ramalho, Tomás Correia, Marília Meira, Cristina Conceição, Severiano R. Silva, Alfredo M. F. Pereira, Joaquim L. Cerqueira

**Affiliations:** 1 Veterinary and Animal Research Centre (CECAV) and Associate Laboratory for Animal and Veterinary Sciences (AL4AnimalS), Department of Animal Science, Universidade de Trás-os-Montes e Alto Douro, Quinta de Prados, 5000-801 Vila Real, Portugal; ssilva@utad.pt (S.R.S.); cerqueira@esa.ipvc.pt (J.L.C.); 2 MED-Mediterranean Institute for Agriculture, Environment and Development and CHANGE-Global Change and Sustainability Institute, Universidade de Évora, Pólo da Mitra, Ap. 94, 7006-554 Évora, Portugal; ersc@uevora.pt (E.C.); cristinaconceicao@uevora.pt (C.C.); apereira@uevora.pt (A.M.F.P.); 3 Department of Animal Science, Universidade de Évora, Pólo da Mitra, Ap. 94, 7006-554 Évora, Portugal; 4 Center for Research and Development in Agrifood Systems and Sustainability (CISAS), Instituto Politécnico de Viana do Castelo, Agrarian School of Ponte de Lima, Rua D. Mendo Afonso, 4990-706 Ponte de Lima, Portugal; 5 Department of Veterinary Medicine, Universidade de Évora, Pólo da Mitra, Ap. 94, 7006-554 Évora, Portugal; 6 Independent Researcher, Malagueira, 7000-407 Évora, Portugal

**Keywords:** abdominal girth, body length, dairy calves, heart girth, hip height, weight scale, withers height

## Abstract

**Simple Summary:**

Calves should be regularly weighed to monitor their growth, which can be an indication of their health and welfare. Pre-weaned calves are particularly vulnerable to health problems as their immune system is still developing. In addition, knowing the calf’s weight can help to provide a more balanced diet and to be more accurate when administering medication. However, there are still many farmers who do not have a scale on their farm or who have limitations in weighing their calves. Therefore, we developed a model using morphometric traits to estimate the weight of Holstein–Friesian pre-weaned calves without the need for a scale, which should be easy to use in most circumstances. Our model used the measurement of heart girth with a simple tape measure and was able to predict the weight of the calf with a mean difference of −1.4 ± 3.24 kg from the actual weight. We did not find any differences between females and males, but we did find differences between farms, which could be due to different genetic lines associated with farm breeding protocols. In addition, a web application was developed to make it easy for farmers to use the developed model. This avoids the use of calibrated weight bands, which are usually calibrated for a wider age range or for beef breeds.

**Abstract:**

Regularly weighing calves helps to assess the efficiency of the rearing period and contributes to animal welfare by allowing more precise feeding and medication application in dairy farming, but many farmers do not weigh their calves regularly. Improving the feasibility of this process is, therefore, important. The use of morphometric measurements has been used to estimate the weight of cattle. However, many studies have focused on adult animals or used a wide age range. As calves experience allometric tissue growth, specific models for certain ranges might be more accurate. Therefore, the aim of this work was to develop a weight estimation model specific for pre-weaned Holstein–Friesian calves using morphometric measurements and to compare the model with another equation previously validated for the same breed with young and adult animals. From four dairy farms, 237 measurements of body weight, heart girth, abdominal girth, hip height, withers height, and body length were taken from Holstein–Friesian male and female calves. Linear and non-linear regression analysis was used to test the relationship between body weight and morphometric measurements, with age, sex, and farm as possible explanatory variables. Selected models were compared with goodness of fit and agreement tests. The final model was able to accurately predict body weight (R^2^ = 0.96) with a mean difference of −1.4 ± 3.24 kg. Differences in the relationship between body weight and morphometric traits were observed between farms, but not between males and females. The genetics of the animal population at farm level may be responsible for this variability and further studies are needed to understand this variability and improve weight prediction models. The developed model was able to perform better in the agreement tests than the previously validated model for Holstein–Friesian animals, suggesting that different equations should be used depending on the growth phase the animal is in. In addition, a web application has been developed to facilitate the use of the developed model by farmers. This avoids the use of calibrated weight bands, which are usually calibrated for a broader age range or for beef cattle.

## 1. Introduction

Regular weighing of calves is an essential management practice for dairy farmers, contributing to animal health and welfare and the overall productivity of the dairy farm. Calf body weight (BW) information can be used for several aspects, such as feeding management practices (e.g., feeding colostrum per BW instead of a standardized volume [1]); calculating medication dosages (e.g., calculating anesthetic and analgesic dosages for disbudding [2] or reducing underdosing caused by incorrect visual weight estimation [3]); to sell animals based on BW, especially for male calves; to test changes in the environment or management practices (e.g., changes in housing system [4]), or to set weight gain targets and assess the rearing period efficiency [5,6]. All these aspects are important for improving calf welfare, financial components, and environmental aspects such as carbon footprint and antimicrobial resistance. 

However, weighing calves may not be practical due to logistical or economic constraints, which may be related to the lack of a reliable scale on the farm or the lack of staff to weigh calves on a regular basis [7] or due to the misperception of visual weight estimation [8]. A survey of calf management practices in the UK found that only 55.6% (120/216) of farmers weighed their calves, and only 31.7% of these farmers used a scale, with the remainder using a weigh tape; moreover, these measurements were taken almost exclusively at birth and weaning, and only 10% weighed their animals on a weekly basis [9]. 

Therefore, if a scale is not available, or if it is available but difficult to use regularly because, for example, the scale is not near the calves’ rearing area and is difficult to move, alternative estimation methods are valuable. Machine vision techniques have been used to reliably estimate bovine BW using automated measurements of body traits [10,11], but such technologies are not yet readily available to most livestock owners and for commercial use [12]. In this manner, linear body measurements or morphometric measurements (MSs) have been used to reliably estimate cattle BW [13,14,15,16]. However, most of these studies have focused on adult animals or in a wide age range, including both younger and older animals in the analysis, with some inconsistencies being reported for younger animals [17]. 

The measurement of heart girth (HG) is the most highly correlated variable with BW in cattle, and generally the addition of a second variable to these models does not compensate for the normally high correlation with HG [13]. For this reason, weight bands were developed so that livestock farmers could estimate the weight of their animals. Heinrichs et al. [18] compared the same equation used in Heinrichs et al.’s [13] study 25 years later and concluded that the previous equation remained valid for Holstein dairy heifers from 1 to 821 d of age. Nevertheless, bovine body traits can change with breed [15,19], age [20,21], and breeding programs over time [18]. Animals have allometric growth of tissues [22], so the relationship between BW and MS is likely to change with age. Sex influences the growth of body tissues [23], so this factor should also be taken into account. It is, therefore, necessary to regularly update these equations, considering breed, sex, and age, to obtain accurate prediction models. Since different phenotypic traits can be found in animals of the same breed [24], these equations should be calculated with individuals from different farms and this factor should be considered when developing predictive models for BW estimation. Furthermore, providing livestock owners with the means to implement these models on a daily basis is equally important.

Therefore, the aim of this study was to develop a new equation specifically for pre-weaned Holstein–Friesian dairy calves and to compare it with the equation proposed by [13], taking into account sex, age, and farm as possible influencing factors. 

## 2. Materials and Methods

### 2.1. Data Collection

The study was approved by the Ethics Committee for Animal Welfare at Universidade Trás-os-Montes e Alto Douro under reference 2664-e-DZ-2023.

The study took place between January 2023 and November 2023 and involved 210 pre-weaned Holstein–Friesian calves, aged 1 to 90 days (160 females and 50 males), from four commercial dairy farms in the Alentejo region of Portugal. During the farm visit, calves aged between 1 and 90 days were weighed using a digital scale (Tru-Test 702, Tru-Test Datamars, Lugano, Switzerland), morphometric measurements (MS) were taken, and sex was recorded. Heart girth (HG) and abdominal girth (AG) were measured with a measuring tape (Comb MAAL, We-Bo, Denmark), and withers height (WH), hip height (HH), and body length (BL) were measured with a hipometer. Twenty-seven animals were weighed twice with a minimum interval of 30 days, as they were part of another study not yet published, resulting in a total of 237 records. All measurements were performed by the same operator on a flat surface with all limbs on the ground, according to [25]. 

**Figure 1:**
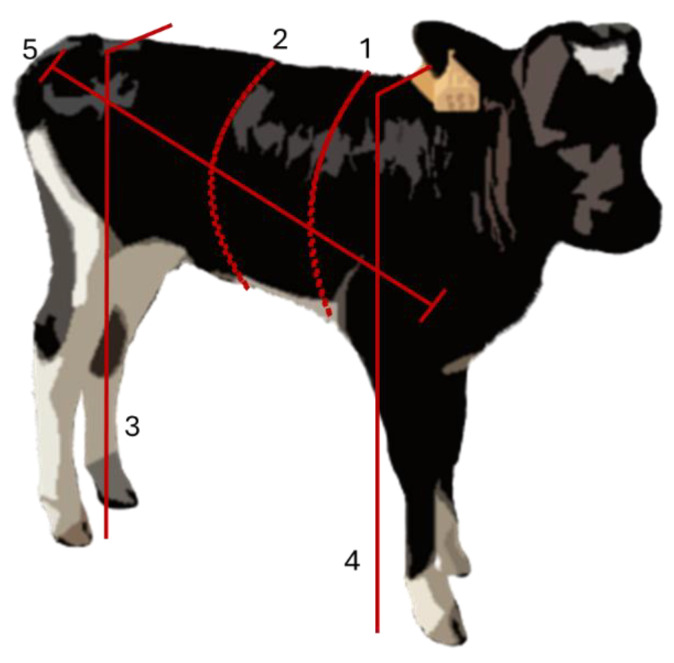
Schematic representation of the morphometric measurements. 1—Heart girth (HG); 2—abdominal girth (AG); 3—hip height (HH); 4—withers height (WH); 5—body length (BL).

HG was measured with a tape around the chest just behind the scapula. AG was measured similarly to HG but at the level of the last rib. WH was measured from the highest point of the withers to the ground and HH from the middle third of the sacrum to the ground. BL was measured as a straight line from the tip of the scapula (the most prominent point of the scapulohumeral joint) to the ischial tuberosity (Figure 1).

### 2.2. Statistical Analysis

All statistical tests were conducted in IBM^®^ SPSS^®^ Statistics, v27 (SPSS).

Variables were tested for normality using graphical analysis and Kolmogorov–Smirnov test; four out of six variables did not show a normal distribution. However, the sum of all variables led to an approximated normal distribution, thus applying the Central Limit Theorem. One calf was removed due to a clear data recording error. Outliers were checked using the studentized deleted residuals and absolute values ≥ 3 were removed. 

To assess initial associations between variables, Spearman’s rank correlation coefficients were calculated. Subsequently, for model creation, several linear and non-linear equations (i.e., simple and multiple) were computed to analyze the relationship between BW as the dependent variable and both MS and age as independent variables. Additionally, an equation used to predict the BW in horses [26] was also tested. This equation can be described as follows:

$$\frac{BL\times {HG}^{2}}{a}$$

where a is fix parameter calculated through an iterative optimization process.

First, the Curve Estimation procedure in SPSS was used to analyze the type of relationship between variables. Linear equations were then calculated using Linear Regression, while non-linear equations were calculated using Non-Linear Regression in SPSS, which incorporates an iterative optimization process to estimate regression parameters. In this process, SPSS starts with a specified initial value for each parameter and then iteratively refines it to minimize the difference between the predicted BW values and the actual data points.

To assess the influence of farm (A, B, C, and D) and sex (female and male) on the explanatory power of the models, we employed dummy coding for these categorical variables. The dummy coded variables and their interaction terms with MS were then incorporated as independent variables in the models. If the interaction between farm/sex and MS showed a statistically effect (*p* < 0.05), indicating that the effect of MS on BW differed depending on farm or sex, separate models were built for each farm–sex combination. Separate models for farm were created using stepwise linear regression with the option to exclude cases listwise for missing values; all potential predictor variables (i.e., MS) were included in the initial model, and the variables of the final model were selected using the stepwise procedure in SPSS.

Final models were selected based on the correlation coefficient (*r*) and the coefficient of determination (R^2^). Predicted BW from the final models was compared with the measured BW with goodness of fit and agreement parameters to select the most appropriate model. The parameters used were the R^2^, the Akaike Information Criterion (AIC) calculated as $n\times \ln(\frac{RSS}{n})+2\times K$, where *K* is the number of parameters of the model, Bland–Altman plots (i.e., plot of the difference between the measured BW and the predicted BW over the mean of these measurements, with ±1.96 SD to define the upper and lower limits of agreement), and the Intraclass Correlation Coefficient (ICC) and their 95% CI to test the reliability between different models (two-way mixed effects, absolute agreement, single, and average measures), using the Reliability Analysis procedure, in SPSS. Student’s *t*-tests were used to assess if the mean difference between measured BW and predicted BW differed from zero (T value = 0). In addition, the equation proposed by Heinrichs et al. (1992) [13] was applied to the database used in this study, and the predicted BW was compared with the final model using the same tests to understand if this alternative was more accurate.

### 2.3. Web Application

**Figure 2:**
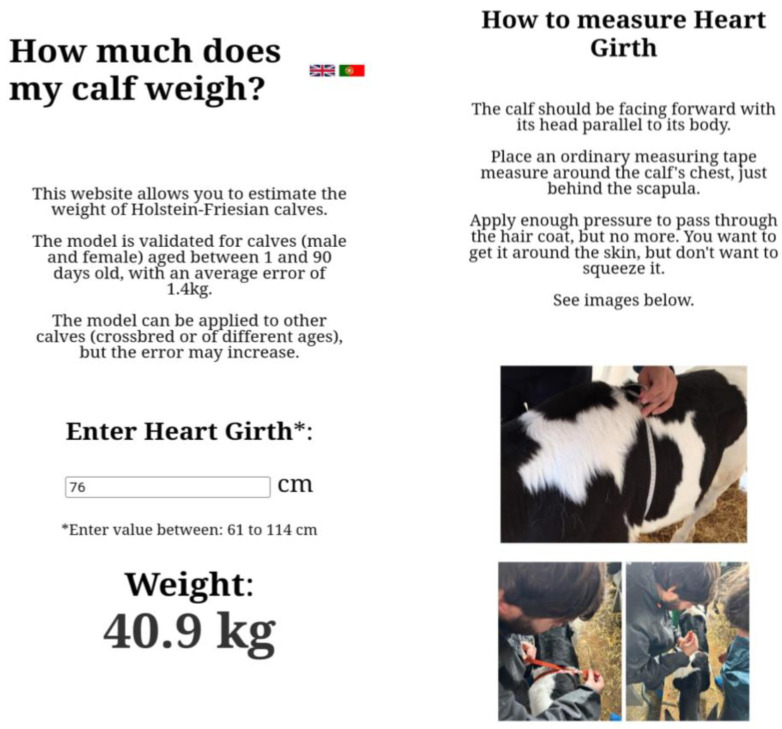
“How much does my calf weigh?” (mobile phone view)—web application developed to allow quick and easy application of the body weight prediction model. Source: https://eloquent-begonia-6dc576.netlify.app/ (accessed on 17 July 2024).

A web application (Figure 2) accessible at https://eloquent-begonia-6dc576.netlify.app/ (accessed on 17 July 2024) leveraging HTML, CSS, and JavaScript programming languages, was developed. The application’s source code is stored on GitHub, facilitating version control and collaborative development, while Netlify serves as the platform for continuous deployment, ensuring the latest updates are readily available.

The web application provides unrestricted access and is compatible with all major web browsers. It offers a user-friendly interface designed to estimate the weight of Holstein–Friesian calves based on their heart girth measurements. It is presented in English and Portuguese languages.

## 3. Results

**Figure 3:**
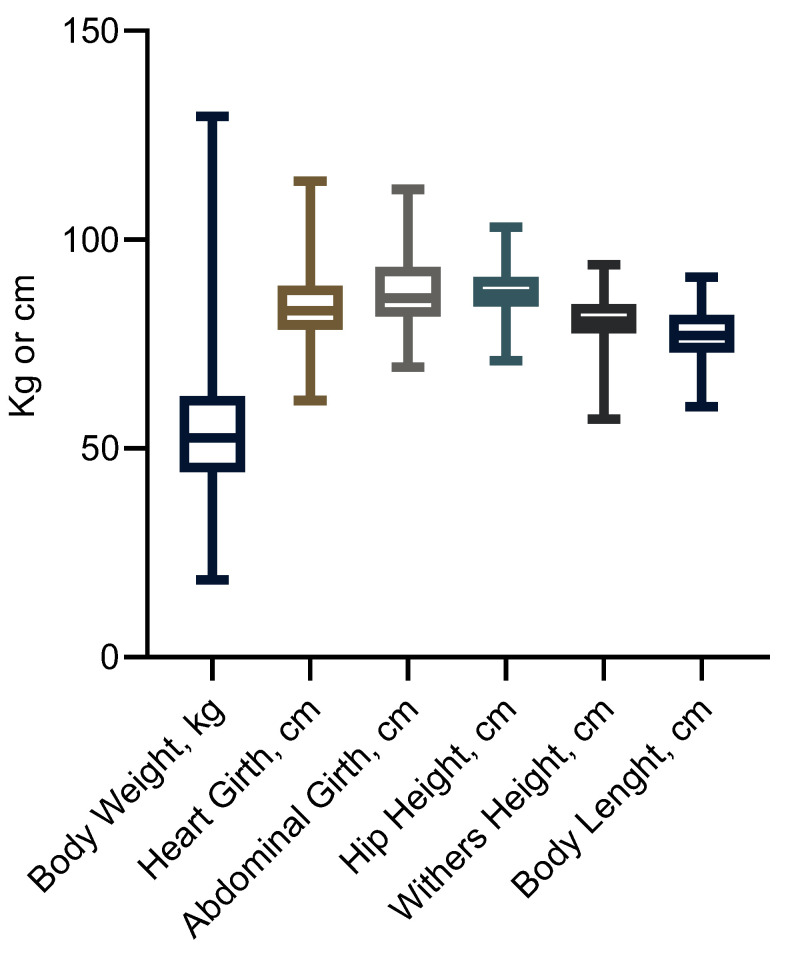
Data distribution of body weight and morphometric measurements.

The age of the calves included in this study and their BWs and MSs are shown in Table 1 and the data distribution is shown in Figure 3. The sample of calves from each farm had a similar age range and both BWs and MSs were similar between farms. From the data distribution shown in Figure 3, BW had the greatest variability between all measurements. This suggests that the relationship between BW and MS is not completely linear.

All MSs were correlated with BW and with each other (*p* < 0.001; Table 2). HG had the best correlation with BW, followed by HH. Plotting BW against BL showed that there were two different relationships (i.e., BL(1) and BL(2)). The BL(1) distribution was best fit by a quadratic curve (n = 129), while the BL(2) distribution was best fit by a linear line (*n* = 58); therefore, two separate correlations were calculated for BL. Age was also positively correlated with BW (0.81, *p* < 0.001). 

Quadratic equations provided higher R^2^ values than linear equations with all MSs (Table 3). In simple linear and quadratic regression analysis, HG had the best fit. Plotting BW against BL showed that there were two different relationships, so two separate models were calculated for BL, which produced different results.

Sex and farm effects were tested only with the HG model as this was the variable most closely associated with BW. Regressing the HG linear equation using sex as a dummy variable showed no significant effect of sex on the BW prediction (*p* = 0.141). However, the interaction between farm and HG was statistically significant (*p* < 0.001), indicating that the predicted values from the HG equation might depend on the farm where the measurements took place. Consequently, separate linear models were created to analyze the relationship between the MSs and BWs for each farm (Table 4). While HG remained the best predictor of BW in every farm, the inclusion of other MS factors improved the model fit depending on the specific farm. 

### 3.1. Model Selection

The model using the horse equation [26] resulted in a considerably lower coefficient of determination (R^2^ = 0.69) and was therefore excluded. Four final models were selected and compared using goodness of fit tests and measurements of agreement to identify the most appropriate one. 

The first model (BW1) consisted of a quadratic equation with HG. The second model (BW2) consisted of a quadratic model with HG and HH. Sex did not significantly affect the predictability of BW using HG; however, females and males were not equally distributed in our sample, so separated models were created as well (i.e., a model for female—BW(f) and a model for male calves—BW(m)). The addition of age to a model with any other MS did not improve the fit and the beta coefficient was always non-significant. Therefore, besides the farm-dependent models, four additional quadratic models were created and presented in Table 5.

### 3.2. Models’ Goodness of Fit and Agreement Tests

**Figure 4:**
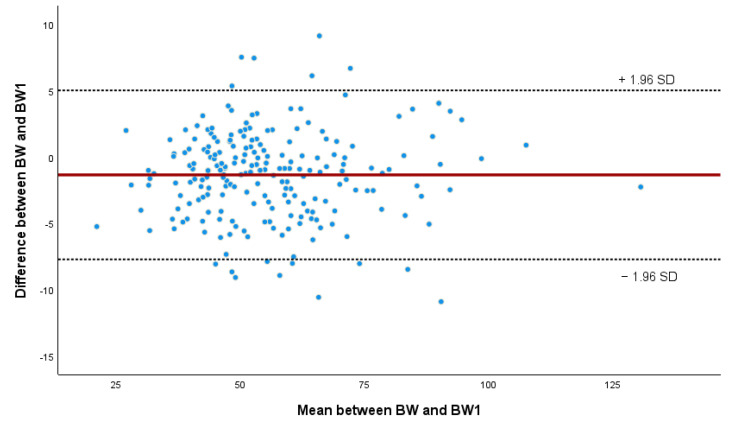
Bland–Altman’s plot of estimated weight (BW1) against measured body weight (BW). Red line represents the mean difference between BW and BW1 and doted lines the upper and lower limits of agreement.

In all models except BW(m), the mean difference was statistically different from zero (*p* < 0.001; Student’s *t*-test), meaning that they were not in perfect agreement with the measured BW and that there is a statistically relevant difference (Table 6). However, when the difference between BW1 and BW was regressed on the mean of BW1 and BW, the β coefficient was close to zero (β = 0.006) and *p* = 0.683, indicating that there is no deterministic bias towards lower or higher BWs for the BW1 model, as can be seen in the Bland–Altman plot (Figure 4). The vast majority (93.2%) of the differences between the two methods fall within the limits of agreement, indicating a good level of agreement between the methods despite the statistical difference. Furthermore, as shown by the graphical distribution of the data (Figure 4), there appears to be no trend in the difference between the BWs at any particular range of BW.

The R^2^ values may not be an adequate measure of the goodness of fit in non-linear models [27]; thus, models were also compared with AIC, Bland–Altman’s plots, and ICC (Table 7). Despite having a lower R^2^ and a higher AIC than BW2, BW1 had a better ICC in both single and average correlations. Additionally, BW2 is more cumbersome since it must use two MS and the increase in R^2^ (1%) was not considered significant.

**Figure 5:**
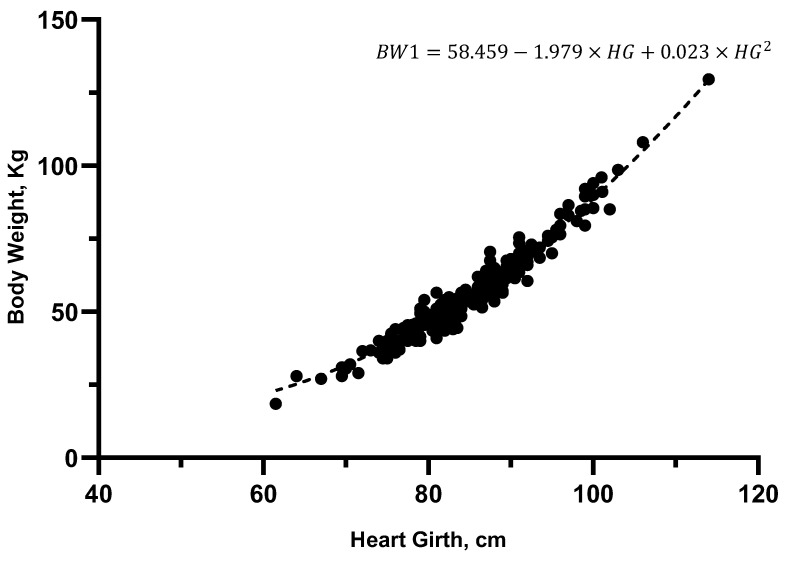
Scatter plot of model BW1, with hearth girth (cm) and body weight (kg), and its respective equation, R^2^ = 0.96.

**Figure 6:**
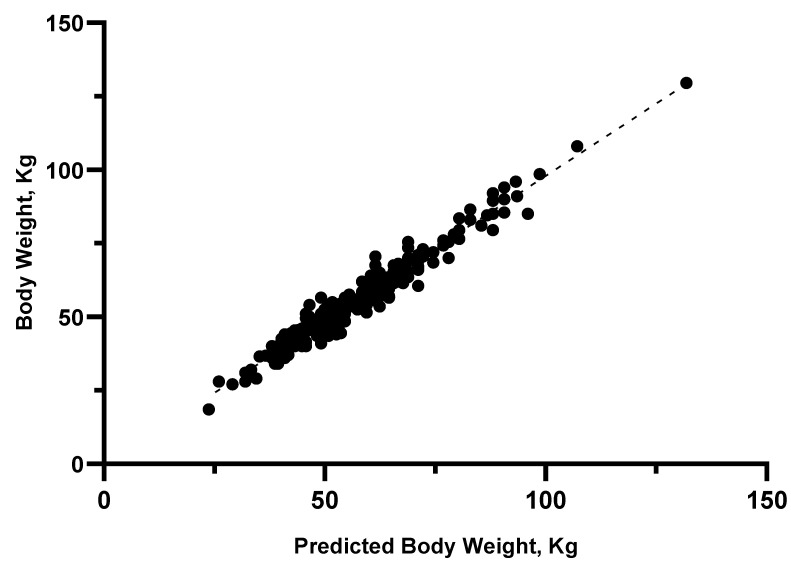
Scatter plot of predicted body weight using model BW1 and measured body weight, R^2^ = 0.96.

The models separated by sex showed a better AIC but a worst ICC than BW1. Therefore, the equation from model BW1 was compared with BW(f) and BW(m) in each respective subsample (i.e., only female or only male calves). In both cases, BW1 was preferred as it had a higher ICC and a lower mean difference between the observed and predicted values (Table 8), which is consistent with the non-significant effect previously reported for sex. Therefore, BW1 was considered the most accurate model. Graphical analysis of this model is shown in Figure 5 and Figure 6. 

### 3.3. Comparison with Equation from Heinrichs et al. (1992)

Using the same tests as in the previous analysis, the equation from [13], hereafter referred to as BW(H92), was applied to our sample and compared with BW1 (Table 9). The results are similar with both models, but while BW1 tended to overestimate the measured BW by 1.4 ± 3.24 kg, BW(H92) tended to underestimate it by 4.7 ± 3.47 kg. The ICC showed a large variability within the 95% CI for BW(H92). 

**Figure 7:**
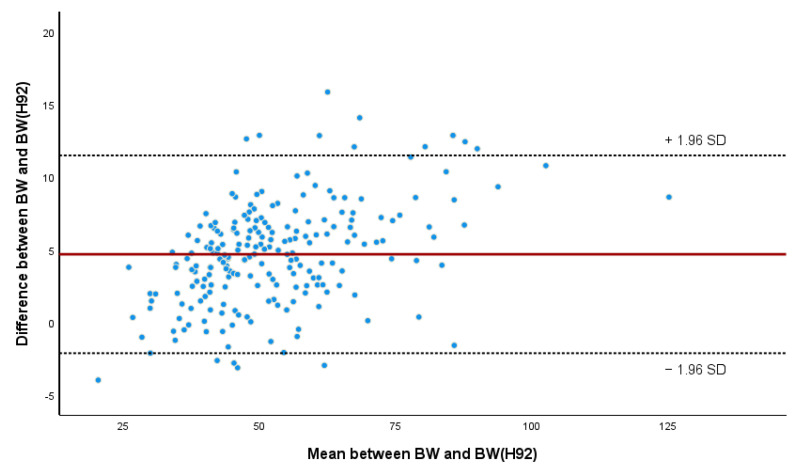
Bland–Altman’s plot of predicted weight (BW(H92)) against measured body weight (BW). Red line represents the mean difference between BW and BW(H92) and doted lines the upper and lower limits of agreement.

The Bland–Altman plot (Figure 7) compares the measured BW with the predicted BW using BW(H92) equation, with 93.6% of values being correctly measured within the 95% confidence interval. However, there seems to be a bias towards underestimating BW as it increases, as can be seen in the Bland–Altman plot.

## 4. Discussion

The use of MSs can be used to predict BW in animals; therefore, several models have been developed over the years. However, due to genetic selection, this relationship can change [18]. In addition, breed and age can also affect this relationship [15,19]; therefore, breed- and age-dependent models are needed [28]. A very strong relationship is needed to reliably predict calf weight to increase precision and production efficiency in calf rearing. Besides reliability, feasibility and robustness are important features for producers to systematically measure calf BW.

As in previous studies [13,19,29], HG was found to be the variable with the best relationship with BW. In our study, besides HG, other MSs showed good results as well, so if HG could not be measured (e.g., in a chute), HH could be measured instead, with an increased error. However, due to the general calmness of this breed and the way animal management is set up, it is usually easy to regularly measure HG in pre-weaned calves. Height may also be more difficult to measure systematically if the ground is uneven, which is not unusual on most farms. In the study by Henrichs et al. [13], the R^2^ values obtained are higher than in this paper, but there could be some collinearity in this case as the same animals were measured seven to thirty-three times. Nevertheless, studies with other breeds, had similar results to our study [8,19,30,31]. García et al. [29] found higher correlations with HG (99.1%) and WH (96.8%) in 104 Holstein heifers aged 4 days to 2 years. 

Few studies have focused on predictive models for MSs in calves and they seem to focus on hoof circumference at the level of the coronary band [32,33,34]. It has been shown that weight estimation with HG has greater variability for heifers with a BW less than 150 kg compared to heavier heifers, with a variability of less than 10% [35]. In our case, the mean variation was 2.55%, similar to the results obtained in [36]. Dingwell et al. [21] also showed that HG measurements in heifers between 3 and 15 months of age were in good agreement with BW, but not in heifers under 3 months of age. In one study, the hoof circumference of newborn beef calves was linearly related to BW (R^2^ = 0.69) [33]. In another study with Holstein and Jersey calves, MSs were related to BW by linear (R^2^ = 0.91) and quadratic (R^2^ = 0.93) regression [34]. Both studies found significant differences between actual and estimated weight as a function of BW range. In the study of Long et al. [34], these differences occurred for calves with a BW lower than 31.3 kg and higher than 44.9 kg. Different goodness of fit were found between different ages (i.e., 2, 8, and 16 weeks), with similar R^2^ values to the present study [17]. It is clear that age plays a significant role in how the BW can be predicted by MS. This finding helps to explain the slightly lower, although still high, correlations in this study, which used calves from a narrower age range, compared to studies using heifers from a wider age range. In addition, some of this variation may be due to abomasum and rumen filling, which should be proportionally more representative in younger than in older animals. 

There is no evidence to suggest the benefits of using sex-specific models in calves of the age in our sample. Although other authors have shown different models according to sex and obtained different results [8,14,37], this does not mean that it is necessarily caused by sex and could be due to the nature of the sample, as we have suggested in this paper, or other factors like age and breed [20,38]. Therefore, the influence of sex on the predictive models for BW should be tested in the presence of other factors (e.g., age and breed). In addition to the differences in MSs between farms, a different relationship between BL and BW was found in one of the farms (farm C). This farm had been visited previously (i.e., 7 months apart) and the distribution of data was similar to that of the other farms. The variability found between farms may be the result of different genetic selection on each farm [13,15]. A difference in BW between farms was found in other work [34], but it was not tested whether ‘farm’ as a factor influenced the relationship between BW and MSs, as we did in the present study, since BW may be different, but the relationship may be identical. However likely this may be, we cannot be sure, and a larger sample size from more farms would provide more information on this topic. Besides genetic selection, considering farm as a factor could imply other influencing factors, like housing conditions, animal handling, and percentage of sick animals, thus further studies are needed. Considering that the farm-specific models developed are not necessarily better than the general models, we decided to use the BW1 for the web application, as it was intended to be used in different farms.

The predicted weights using our model BW1, or BW(H92) were similar, but our model seems to be more appropriate for our sample. BW(H92) had a wider age range, and since the growth tissues of calves are allometric, it seems more advantageous to have a different predictive model for younger ages. Using an analysis that includes age as a factor of variation is difficult to implement in a weigh tape, so with a simple tape measure and a mobile application we can use more appropriate equations that include the age of the animal. There are several calibrated HG bands, but they can vary in the equation used and are not specific to dairy calves. Thus, the web application developed can overcome this limitation, since a regular tape measure can be used to take the HG and then the result is given using a digital platform (e.g., mobile phone and laptop). We believe that this method can increase the accuracy and feasibility of the measurements and improvements to the model can be made in further studies.

## 5. Conclusions

In this study, we proposed a model for estimating body weight from morphometric measurements in pre-weaned Holstein–Friesian calves aged 1 to 90 days. In addition, we developed a web application to increase uniformity and feasibility at farm level. The results indicate that calf body weight can be reliably estimated using heart girth measurements. Dairy farmers without access to a weighing scale can use this model to reliably assess the efficiency of the pre-weaning period or to provide colostrum, milk, starter, or medication based on weight. The relationship between morphometric measurements and body weight may depend on the genetic make-up of the population. Therefore, further studies to validate this model in different farms would be beneficial to increase its robustness.

## Figures and Tables

**Table 1:** Descriptive statistics (mean ± standard deviation) for age, body weight, and morphometric measurements across farm.

Source	N ^1^	Age, Days	BW, kg	HG, cm	AG, cm	HH, cm	WH, cm	BL, cm
**Farm A**	43	35.6 ± 15.98	56.3 ± 11.23	85.7 ± 6.04	91.3 ± 8.90	86.8 ± 4.66	82.1 ± 4.28	77.7 ± 5.67
**Farm B**	50	27.8 ± 19.34	56.9 ± 16.02	84.6 ± 7.52	93.4 ± 10.44	88.7 ± 5.93	82.6 ± 4.91	77.4 ± 6.74
**Farm C**	90	32.7 ± 22.32	50.8 ± 11.14	82.1 ± 6.63	84.1 ± 7.02	86.3 ± 5.13	78.0 ± 7.15	77.1 ± 4.74
**Farm D**	54	24.4 ± 27.77	59.7 ± 22.38	85.7 ± 10.1	89.9 ± 9.14	88.8 ± 7.03	83.2 ± 5.94	77.3 ± 8.52

^1^ Number of weight measurements, other measurements besides HG were not measured in all calves. BW—Body weight; HG—heart girth; AG—abdominal girth; HH—hip height; WH—withers height; BL—body length.

**Table 2:** Spearman’s correlations between body weight and morphometric measures in pre-weaned Holstein–Friesian calves.

	BW	HG	AG	HH	WH
**HG**	0.97				
**AG**	0.87	0.86			
**HH**	0.88	0.85	0.72		
**WH**	0.81	0.79	0.77	0.81	
BL(1) *	0.88	0.86	0.77	0.79	0.78
BL(2) *	0.94	0.93	0.78	0.82	0.84

All correlations where significant at *p* < 0.001. BW—Body weight; HG—hearth girth; AG—abdominal girth; HH—hip height; WH—wither height; BL—body length. * BL was divided into two, since two very distinct relationships were found in this measurement.

**Table 3:** Regression coefficients of linear and quadratic equations for pre-weaned Holstein–Friesian calves body weight estimated from morphometric measures.

Measurement	Intercept	Linear	Quadratic	*r*	R^2^	N
HG	−108.660	1.943		0.97	0.94	234
	58.459	−1.979	0.023	0.98	0.96	
AG	−55.298	1.248		0.89	0.79	168
	−86.236	1.943	−0.004	0.89	0.80	
HH	−143.701	2.273		0.90	0.81	214
	149.210	−4.469	0.039	0.91	0.83	
WH	−98.606	1.901		0.84	0.70	219
	193.423	−5.555	0.047	0.86	0.75	
BL(1) *	−97.034	2.009		0.88	0.78	116
	181.688	−5.274	0.047	0.90	0.81	
BL(2) *	−50.216	1.175		0.96	0.93	61
	−95.090	2.304	−0.007	0.97	0.93	

All parameters where significant at *p* < 0.001. HG—Hearth girth; AG—abdominal girth; HH—hip height; WH—wither height; BL—body length; *r*—correlation coefficient; R^2^—coefficient of determination; N—number of measurements * BL was divided into two models, since two distinct relationships were found with this measurement.

**Table 4:** Multiple linear regression models for pre-weaned Holstein–Friesian calves body weight estimated from morphometric measurements for each farm.

β Coefficient	N	Intercept	HG	AG	WH	HH	BL	*r*	R^2^
**Farm A**	42	−112.739	1.472 ***		0.524 *			0.96	0.95
**Farm B**	28	−197.34	1.115 ***	0.207 *	1.701 ***			0.99	0.97
**Farm C**	90	−94.214	1.209 ***	0.283***		0.253 *		0.97	0.95
**Farm D**	43	−141.146	1.842 ***			0.485 **		0.99	0.97

*** *p* < 0.001, ** *p* < 0.01, * *p* < 0.05. HG—Hearth girth; AG—abdominal girth; HH—hip height; WH—wither height; BL—body length; r—correlation coefficient; R^2^—coefficient of determination; N—number of measurements.

**Table 5:** Selected models for estimating body weight from morphometric measurements. Model identification and their respective equation and description are provided.

Model ID	Equation	Description
BW1	$BW=58.459-1.979\times HG+0.023\times {HG}^{2}$	simple quadratic regression with HG (n = 234)
BW2	$BW=62.449-1.979\times HG+0.026\times {HG}^{2}-0.284\times HH+0.01\times {HG}^{2}-0.011\times (HG\times HH)$	multiple quadratic regression with HG and HH (n = 214)
BW(f)	$BW=71.128-2.28\times HG+0.025\times {HG}^{2}$	simple quadratic regression with HG, females only (n = 185)
BW(m)	$BW=14.756-0.946\times HG+0.017\times {HG}^{2}$	simple quadratic regression with HG, males only (n = 49)

**Table 6:** Characteristics of the measured body weight (BW) and the predicted body weight (BWp) for each corresponding model.

Model	BW ^1^	BWp ^1^	BW-BWp ^2^	*p* Value	N
**BW1**	55.0 ± 15.65	56.3 ± 15.56	−1.4 ± 3.24	<0.001	234
**BW2**	55.8 ± 15.77	52.9 ± 14.99	2.9 ± 2.97	<0.001	218
**BW(f)**	55.8 ± 16.43	59.1 ± 16.80	−3.2 ± 2.95	<0.001	185
**BW(m)**	51.8 ± 11.90	52.7 ± 11.27	−0.9 ± 4.19	0.137	49

^1^ Mean ± standard deviation; ^2^ mean difference; N—number of measurements.

**Table 7:** Goodness of fit and agreement tests between models.

Model	*r*	R^2^	AIC	ICC (Single)	ICC (Average) *
**BW1**	0.98	0.96	575.2	0.98 [0.96–0.98]	0.99 [0.98–0.99]
**BW2**	0.98	0.97	495.6	0.97 [0.78–0.99]	0.92 [0.87–0.99]
**BW(f)**	0.98	0.97	397.8	0.97 [0.70–0.99]	0.98 [0.82–0.99]
**BW(m)**	0.93	0.88	149.1	0.93 [0.88–0.96]	0.97 [0.94–0.98]

*r*—Correlation coefficient; R^2^—coefficient of determination; AIC—Akaike information criterion; ICC—intraclass correlation coefficient. AIC calculated as: $n\times \ln(\frac{RSS}{n})+2\times K$, where K is the number of parameters. * ICC of the average between measured body weight and predicted body weight. The ICC 95% CI are shown between square brackets.

**Table 8:** Characteristics of the measured body weight (BW) and the predicted body weight (BWp) and Intraclass Correlation coefficients for female and male data using the BW1 model.

Database	Model	BW ^1^	BWp ^1^	BW-BWp ^2^	*p*-Value	ICC (Single)	ICC (Average) ^3^	N
**Female**	BW1	55.8 ± 16.43	57.4 ± 16.41	−1.6 ± 2.90	<0.001	0.99 [0.96–0.99]	0.99 [0.98–0.99]	185
**Male**	BW1	51.8 ± 11.9	52.2 ± 11.05	−0.4 ± 4.22	<0.46	0.93 [0.89–0.96]	0.97 [0.94–0.98]	49

^1^ Mean ± standard deviation; ^2^ mean difference; ^3^ ICC of the average between measured body weight and predicted body weight. ICC—Intraclass correlation coefficient; N—number of measurements.

**Table 9:** Comparison of the measured body weight (BW) with the predicted body weight (BWp) using BW1 and BW(H92) [13] equations.

Model	BW ^1^	BWp ^1^	BW-BWp ^2^	ICC (Single)	ICC (Average) ^3^	N
**BW1**	55.0 ± 15.65	56.3 ± 15.56	−1.4 ± 3.24 *	0.98 [0.96–0.98]	0.99 [0.98–0.99]	234
**BW(H92)**	55.0 ± 15.65	50.3 ± 14.01	4.7 ± 3.47 *	0.93 [0.30–0.98]	0.96 [0.45–0.99]	234

^1^ Mean ± standard deviation; ^2^ mean difference; ^3^ ICC of the average between measured body weight and predicted body weight. N—number of measurements; * *p* < 0.001.

## Data Availability

Data is contained within the article. The original contributions presented in the study are included in the article, further inquiries can be directed to the corresponding author.
